# Cone inputs to murine striate cortex

**DOI:** 10.1186/1471-2202-9-113

**Published:** 2008-11-14

**Authors:** Björn Ekesten, Peter Gouras

**Affiliations:** 1 Department of Clinical Sciences, Swedish University of Agricultural Sciences, Uppsala, Sweden; 2 Department of Ophthalmology, Columbia University, New York, NY, USA

## Abstract

**Background:**

We have recorded responses from single neurons in murine visual cortex to determine the effectiveness of the input from the two murine cone photoreceptor mechanisms and whether there is any unique selectivity for cone inputs at this higher region of the visual system that would support the possibility of colour vision in mice. Each eye was stimulated by diffuse light, either 370 (strong stimulus for the ultra-violet (UV) cone opsin) or 505 nm (exclusively stimulating the middle wavelength sensitive (M) cone opsin), obtained from light emitting diodes (LEDs) in the presence of a strong adapting light that suppressed the responses of rods.

**Results:**

Single cells responded to these diffuse stimuli in all areas of striate cortex. Two types of responsive cells were encountered. One type (135/323 – 42%) had little to no spontaneous activity and responded at either the on and/or the off phase of the light stimulus with a few impulses often of relatively large amplitude. A second type (166/323 – 51%) had spontaneous activity and responded tonically to light stimuli with impulses often of small amplitude. Most of the cells responded similarly to both spectral stimuli. A few (18/323 – 6%) responded strongly or exclusively to one or the other spectral stimulus and rarely in a spectrally opponent manner.

**Conclusion:**

Most cells in murine striate cortex receive excitatory inputs from both UV- and M-cones. A small fraction shows either strong selectivity for one or the other cone mechanism and occasionally cone opponent responses. Cells that could underlie chromatic contrast detection are present but extremely rare in murine striate cortex.

## Background

The murine retina contains middle (M) and ultra-violet (UV) sensitive cone opsins, which could provide the mouse with a form of colour vision. These two cone opsins, however, are distributed in different regions of the retina; the M- and UV-cone opsins are more common in the superior and ventral retina, respectively, which complicates comparing their responses in the same areas of visual space, a logical requirement for colour vision. In addition, many murine cones seem to express both M- and UV-cone opsins [1,2], which is also a handicap for colour vision. Nevertheless behavioural studies indicate that mice have colour vision [3] and anatomical evidence exists for UV cone specific bipolar cells [4], another requirement for colour vision. We have also found evidence that some retinal ganglion cells receive inputs exclusively from UV-cones [5] supporting the latter anatomical findings. Therefore the question of colour vision in the mouse deserves further study. To do this, we have recorded responses from single neurons in murine visual cortex to determine whether there is any unique selectivity for cone inputs at this higher region of the visual system that would support the possibility of colour vision in mice.

## Results

### Phasic cells

**Figure 1:**
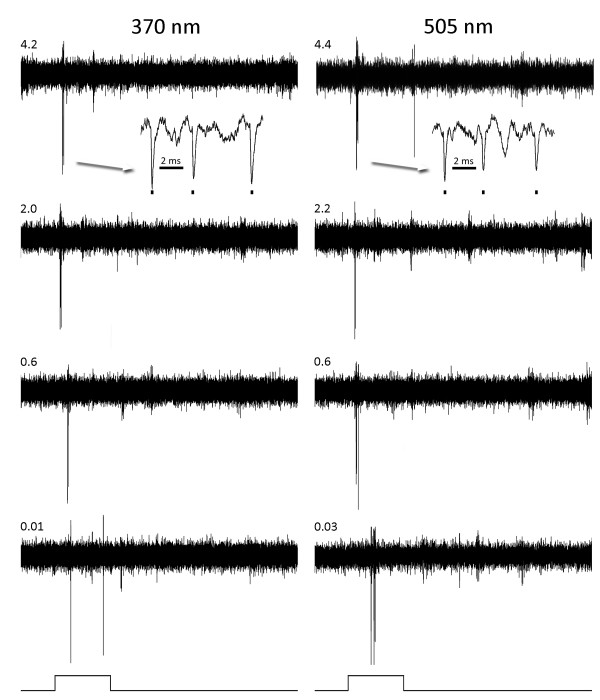
**An on phasic cell**. Responses of an on phasic cell in murine striate cortex to different energies of stimulation with 370 nm (left) and 505 nm (right) light. Inserts (arrows) show the responses on a faster time base and marks below indicate the impulses of the unit studied. The numbers to the left of each trace show the energy in W/m^2 ^of the light pulses, which are 0.4 seconds in duration. Positive is an upward deflection in this and subsequent figures.

**Figure 2:**
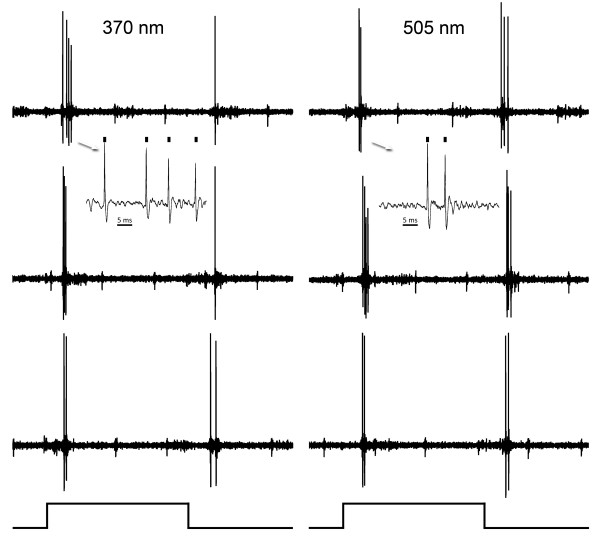
**An on-off phasic cell**. Responses of an on-off phasic cell, responding similarly to both wavelengths at maximal stimulation. Three responses to the same stimulus are illustrated in each column to show consistency. Inserts (arrows) show responses on a faster time base and marks above indicate the impulses of the unit studied. The duration of the light stimulus (below) is 0.5 seconds in duration.

We encountered 323 cells in all areas of striate cortex. 301 (93%) could be driven by our diffuse light stimulus to the contra-lateral eye. In some cases we could also affect the same cell by stimulating the ipsi-lateral eye. There were two varieties of cell responses. One type of cell had little to no spontaneous activity and responded with a few impulses of high frequency either at the on and/or the off phase of the stimulus (42%). These units tended to have large amplitudes. Figure 1 shows such a cell responding to both 505 and 370 nm stimuli at different energies of light stimulation from supra-threshold to approximately threshold levels. This cell responded about equally to both wavelengths at maximum stimulation. The latencies of these responses were about 35 milliseconds to the strongest stimuli, but latencies increased as the energy of stimulation was reduced. With strong stimuli there is an off as well as an on response, especially to 505 nm stimulation. We judged this cell to respond approximately equally to the two spectral stimuli. Figure 2 shows such a typical phasic cell responding to both the on and the off phase of the light stimulus; three responses to the same stimulus are arranged in each column to show consistency. This cell also exhibits approximately equal responsiveness to both wavelengths.

### Tonic cells

**Figure 3:**
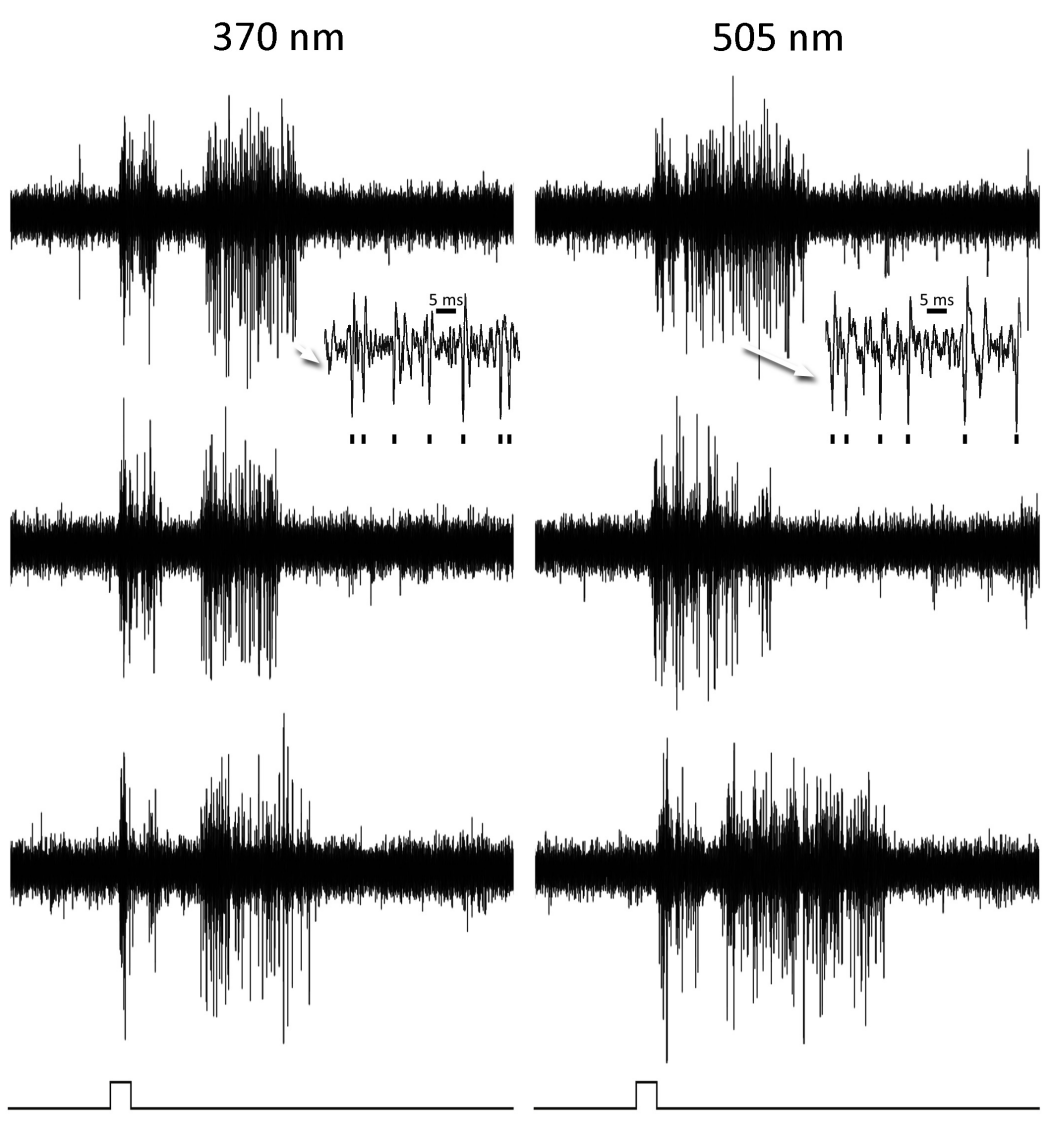
**An on-off tonic cell**. Responses of an on-off tonic cell in murine striate cortex to maximal stimulation with 370 (left) and 505 (right) nm. Three responses to the same stimulus are shown in each column to illustrate consistency. Inserts (arrows) show responses on a faster time base and marks below indicate the impulses of the unit studied. The duration of the light pulse (below) is 0.1 seconds. This cell responds similarly to both wavelengths.

Another type of cell had more spontaneous activity and tended to respond tonically (Figure 3). These cells, were slightly more frequently observed (51%) than the phasic cells and usually had impulses of smaller amplitude. Most of these cells also showed no strong preference for either wavelength, as exemplified by the responses of Figure 3. The burst-like responses shown in this figure were often seen in tonic units with ketamine-xylazine anesthesia. Tonic and phasic cells could frequently be recorded simultaneously in the same penetration implying that they do not target different local areas of striate cortex.

There were about equal numbers of tonic and phasic cells and many cells showed responses to both the on and the off phase of stimulation (Table 1). Very few cells encountered could not be driven. It is understood that the frequency of encountering certain cells rather than others can depend on the characteristics of the microelectrodes being used.

**Table 1:** Physiological characteristics of cells in striate cortex

	**On**	**Off**	**On-off**	**Opponent**	**Undrivable**	*Total*
**Tonic**	68	10	87	*2*	n/a	*166*
**Phasic**	70	15	48	*1*	n/a	*135*
**Undrivable**	n/a	n/a	n/a	*n/a*	n/a	*22*
*Total*	*138*	*25*	*135*	*3*	*22*	*323*

Most cells could be characterized by these physiological characteristics. Undrivable cells were detected by their spontaneous activity and their relatively large amplitude, which led to repeated attempts to detect a response to our light stimuli.

### Cone selective cells

**Figure 4:**
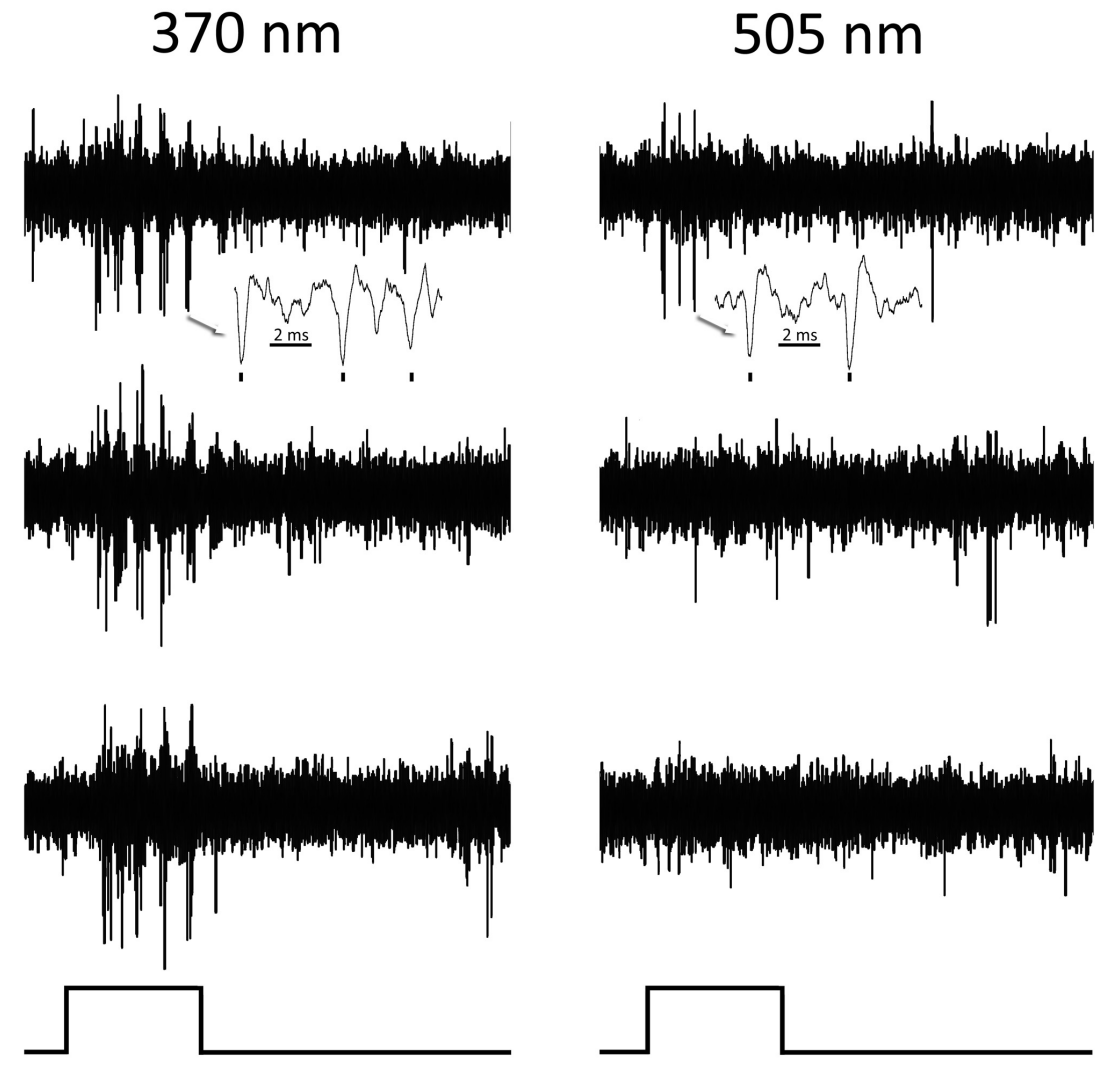
**An on tonic cell responding more strongly to 505 nm**. Responses of an on-tonic cell in murine striate cortex to maximal stimulation with 370 (left) and 505 (right) nm. Three responses to the same stimulus are shown to illustrate consistency. Inserts (arrows) show responses on a faster time base and marks below indicate the impulses of the unit studied. The duration of the light pulse (below) is 0.4 seconds. This cell responds much more strongly to 370 than to 505 nm light.

**Figure 5:**
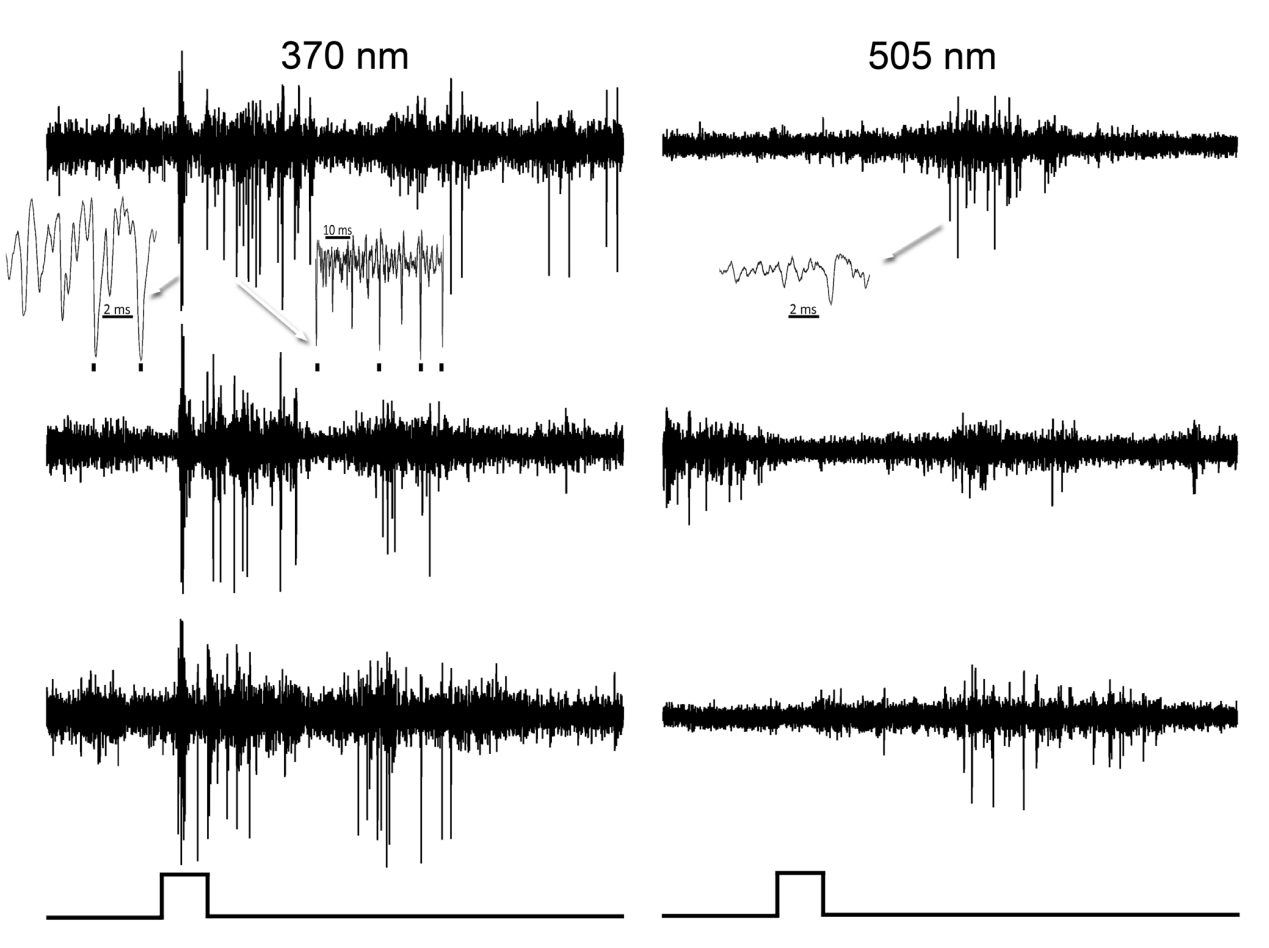
**A tonic cell responding exclusively to 370 nm**. Responses of an on-off tonic cell in murine striate cortex to maximal stimulation with 370 (left) and 505 (right) nm. Three responses to the same stimulus are shown to illustrate consistency. Inserts (arrows) show responses on a faster time base and marks below indicate the impulses of the unit studied. The duration of the light pulse (below) is 0.1 seconds. This cell responds only to 370 nm stimuli.

**Figure 6:**
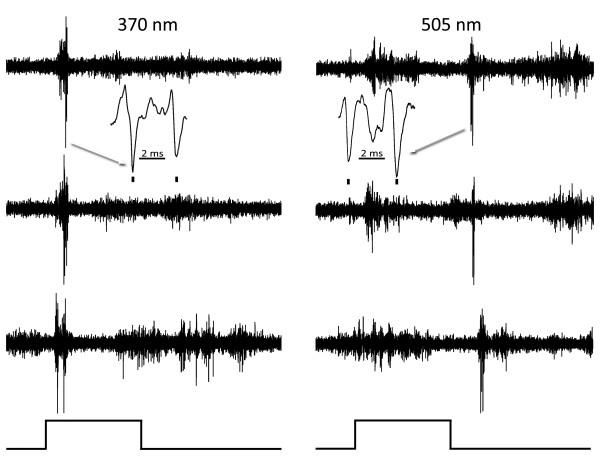
**A phasic, cone opponent cell**. Responses of a phasic cell in murine striate cortex, exhibiting cone opponent behavior to maximal stimulation with 370 (left) and 505 (right) nm. Three responses to the same stimulus are shown to illustrate consistency. Inserts (arrows) show responses on a faster time base and marks below indicate the impulses of the unit studied. The duration of the light pulse (below) is 0.3 seconds in duration.

**Figure 7:**
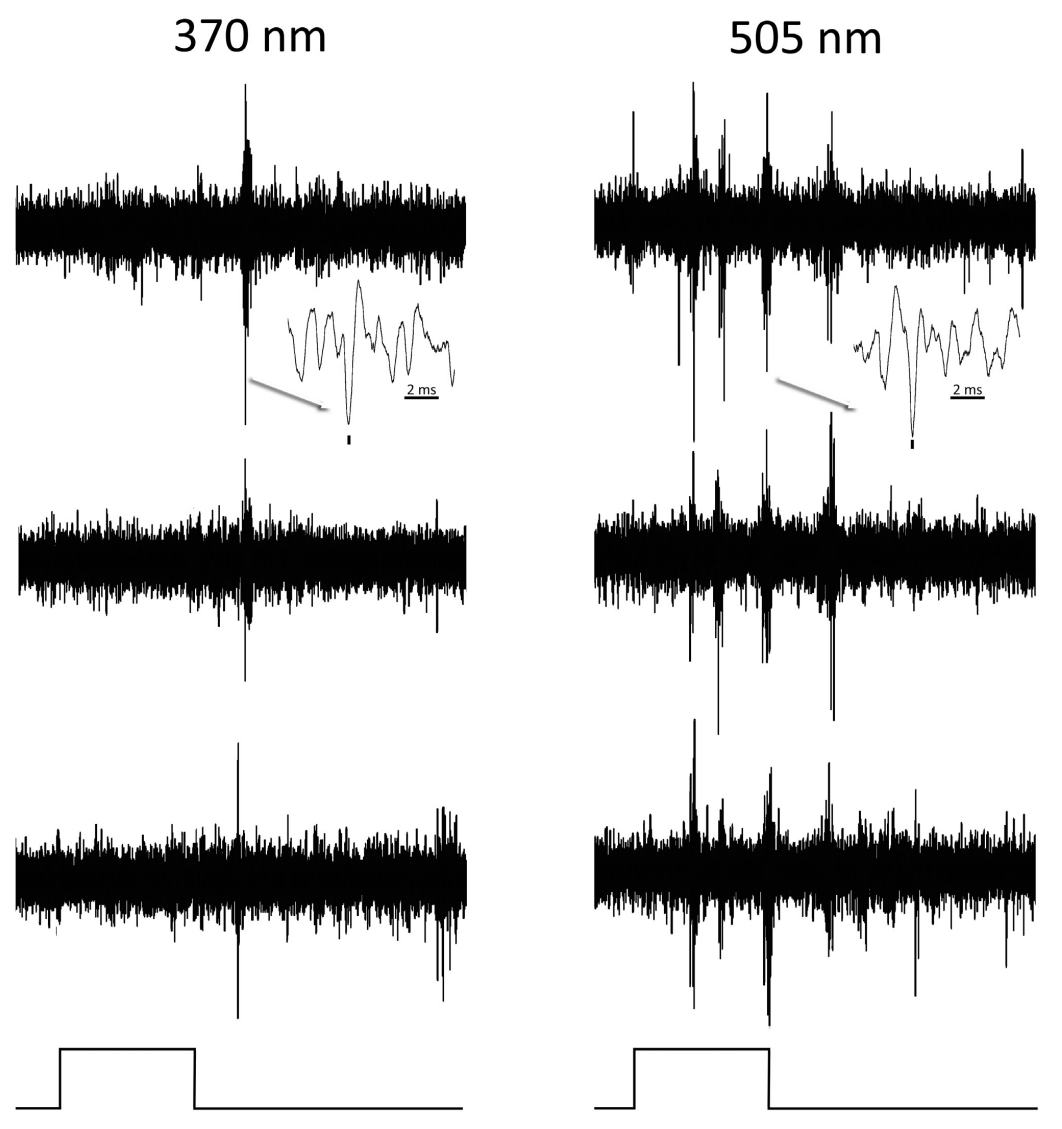
**A tonic, cone opponent cell**. Responses of a tonic cell in murine striate cortex exhibiting cone opponent behaviour to maximal stimulation with 370 (left) and 505 (right) nm light. Three responses to the same stimulus are shown to illustrate consistency. Inserts (arrows) show responses on a faster time base and marks below indicate the impulses of the unit studied. The duration of the light pulse (below) is 0.4 seconds.

Some cells showed a very unequal response to the two wavelengths. Figure 4 shows a tonic cell responding more strongly to 370 than to 505 nm. Figure 5 shows another example where there is no response to 505, but a strong tonic response to 370 nm. Figure 6 shows a phasic cell with cone opponent behaviour. The cell responds at on to 370 and at the off to 505 nm. Figure 7 shows an example of a tonic cell with an opposite cone opponent response. This cell responds weakly at off to 370 and strongly at on to 505 nm. Reducing the energy of stimulation weakened these responses but did not change the opponent polarity.

**Figure 8:**
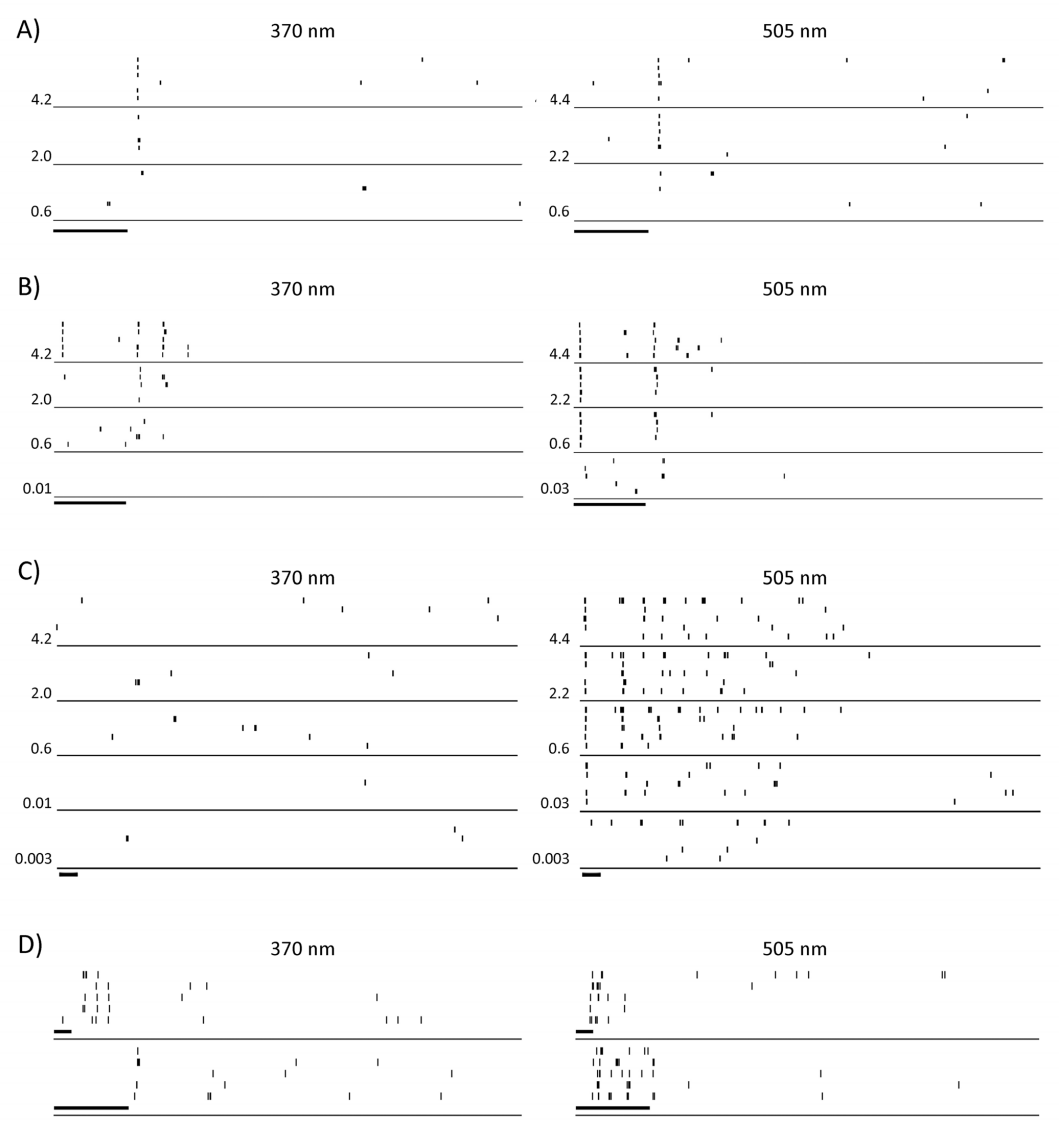
**Discriminated responses from four types of cells in striate cortex**. Dot graphs of four cells in murine striate cortex. **A**) Responses of an off-phasic cell to different energies of stimulation from maximal to threshold levels. The numbers at the left of each trace indicate the energy of stimulation in W/m^2 ^in all panels. **B) **Responses of an on-off phasic cell more strongly driven by 505 nm than 370 nm. **C) **An on-off tonic cell only responding to 505 nm. **D) **A cone opponent tonic cell responding to short and long duration stimuli. This cell responds at off to 370 nm and at on to 505 nm. The duration of light stimulation is shown by the thick bars below the traces (short bars 100 ms; long bars 400 ms).

We also applied a window discriminator to the digital records to obtain more quantitative examples of responsiveness. Figure 8A shows a dot graph of a phasic cell that responds at the off phase of the light pulse and equally to both wavelengths. Figure 8B shows another phasic cell which responds at both the on and the off phase of the light pulse and stronger to 505 than to 370 nm stimulation. Changing the energy of stimulation to threshold levels had little effect on the polarity of the responses. Figure 8C shows a tonic cell that responds only to 505 nm stimulation and not to 370 nm. Figure 8D shows a tonic cell that responds at the off phase of the light pulse to 370 nm and at the on phase to 505 nm. Longer duration light pulses do not change the cone opponent behaviour of this cell and illustrate the tonic nature of the response. Therefore cells in murine striate cortex can show strong selectivity for M- and UV-cone inputs and cone opponent behaviour, but such cells are very rare.

**Figure 9:**
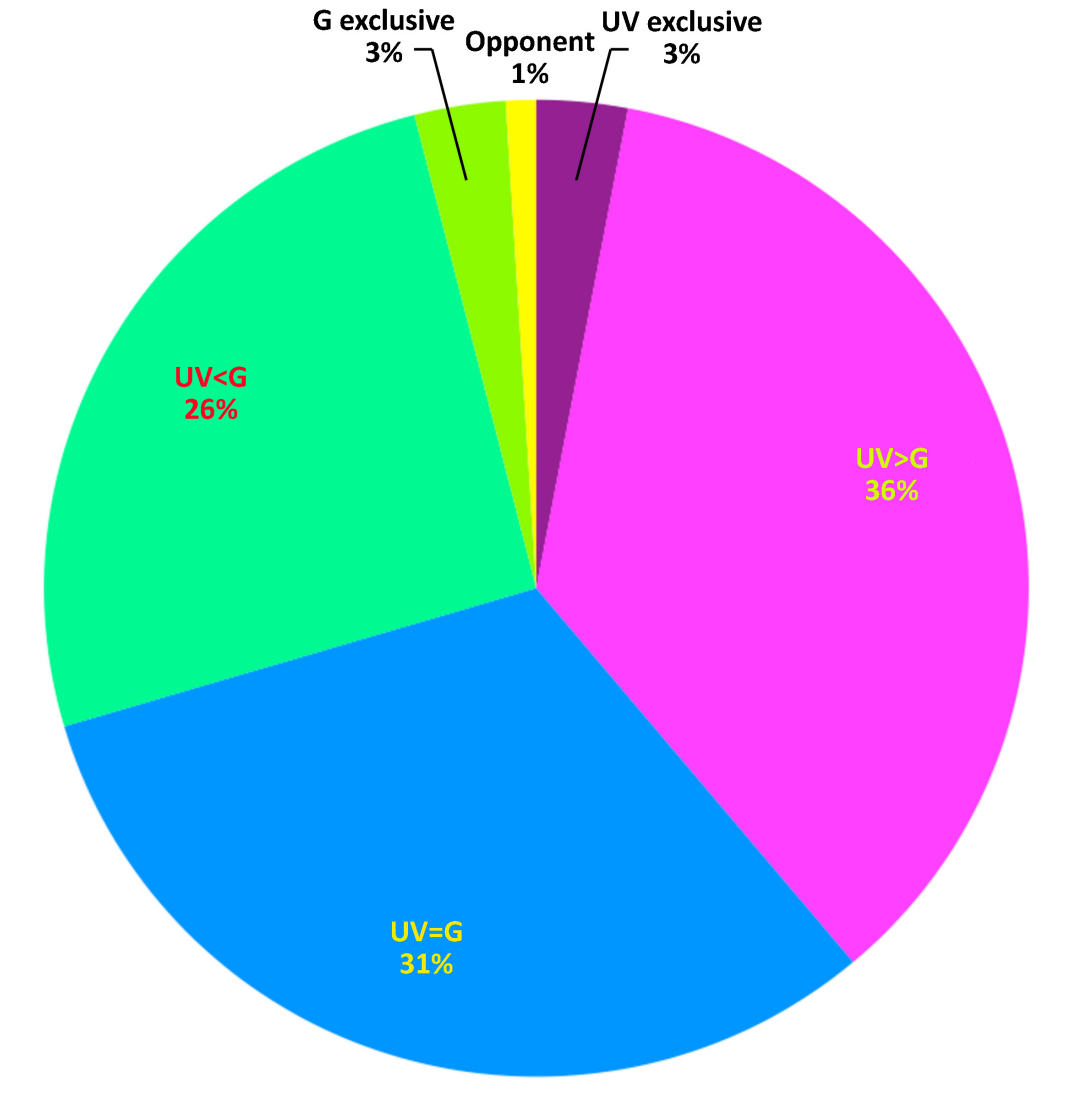
**Responses to different chromatic stimuli**. The distribution of cells according to the strength of their responses to 370 nm (UV) and 505 nm (G). Cone selective and cone opponent cells comprise a small fraction.

Cells showing stronger responses to 370 nm than to 505 nm stimuli were the most common (36%; Figure 9), whereas cells with equal preference to the two wavelengths comprised 31%. Twenty-six percent of the cells showed stronger responses to 505 nm than to 370 nm. A very small group of cells responded only to 505 or only to 370 nm and an even smaller percentage showed cone opponent behaviour.

### Cortical topography of cone inputs

**Figure 10:**
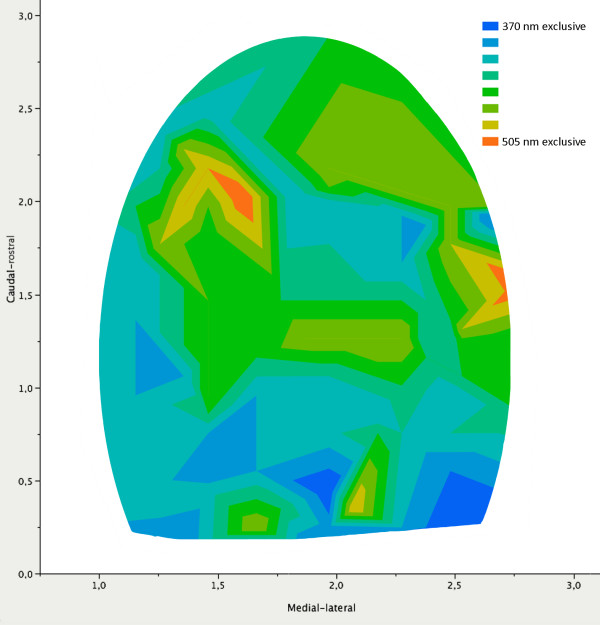
**Cortical topography of cone inputs**. In this contour plot, areas with the same average strength of the two cone inputs have the same colour. The colour coding goes from orange (an area where responses were only driven by the 505 nm) to blue representing an area with cells exclusively responding to the 370 nm. The vertical axis shows the caudal-rostral direction and the horizontal axis shows the medial-lateral direction of the striate cortex perimeter in millimetres.

In almost all areas, cells responded to both wavelengths in a similar fashion although there was a tendency for cells located in the caudal-lateral portion of striate cortex to respond more strongly to 370 than to 505 nm and vice versa in the rostral portion (Figure 10). The three cells showing cone opponent behaviour were located in the caudal or intermediate part of the striate cortex.

We estimated the depth at which we encountered cells in each penetration by the reading of the micrometer drive. This is not an accurate method because it is often difficult to know exactly when the microelectrode has just entered the cortex and the angle of penetration is not always perpendicular to the cortical layers. Our overall estimate was that most of the cells encountered were in layers 2, 3 and 4.

## Discussion

Our results show that all areas of striate cortex have cells that respond to diffuse light stimulation. This presumably reflects reports that a high percentage of neurons in murine striate cortex do not show orientation selectivity [6-9]. These may be the cells that we studied, although it is possible that murine cortical cells could respond to full field stimuli and also show orientation selectivity to slits or bars. Whether the cell bodies of these cells were located in striate cortex is assumed and strengthened by occasional binocular responses. The main goal of our study was to determine if cells with exclusive selectivity for one cone mechanism exist in striate cortex and therefore this distinction as to whether a cell was an incoming geniculate axon or a cortical cell was not critical to this goal.

Our results reveal that most cells in murine striate cortex respond similarly to both wavelengths implying that they are receiving inputs from both UV- and M-cones. We assume that 505 nm light only affects M- and not UV-cone opsins, because the UV-cone opsin absorbs no light in this region of the spectrum [10]. We also assume that 370 nm light affects these cells through UV-cones and not through the beta band of M-cone opsin. This assumption is strengthened by the fact that the sensitivity for 370 nm tended to be as great or greater than it was for 505 nm, which would not be the case if we were affecting these cells by the much less sensitive beta-band of M-cone opsin. A higher sensitivity to ultra-violet than to mid-spectral light agrees with recordings of the murine electroretinogram and retinal ganglion cell responses [3,11]. The strong responsiveness to both wavelengths implies that most cells in murine striate cortex are using achromatic rather than colour contrast for vision. It also implies that the chromatic aberration inherent in ultra-violet versus mid-spectral images can be handled by the murine visual system. Chromatic aberration may be less of a problem due to greater depth of focus inherent in the small eye of the mouse and the structural properties of the lens [12,13].

That the effectiveness of these two wavelengths was relatively similar throughout striate cortex was surprising in comparison to the representation of these two cone mechanisms across the retina [14]. We expected that the caudal part of striate cortex, reflecting the inferior retinal projection, would be more obviously dominated by UV-cone inputs and the rostral part more representative of M-cones. This difference was not apparent, although there was a tendency for UV-cone strength to be greater caudally. Perhaps this lack of representation of cone mechanisms according to their retinal representation reflects co-expression of M-cone opsin in many UV-cones. Another possibility could be that our full field stimulus drives a cell system, which has a large projection area in striate cortex and tends to receive an input from both cone mechanisms. This more global system may inhibit a more local system that reflects the retinal anatomy more closely.

There were only a few cells that responded strongly or only to one of these two wavelengths and very few that responded in an opponent fashion. It would be valuable to have more information about the receptive field organization of these rare cone opponent cells, although this is somewhat difficult to do with ultra-violet stimuli that the investigators cannot see. The rarity of such cone selective cells could reflect the fact that chromatic contrast has an inherently lower spatial resolution than achromatic contrast and therefore may involve fewer cortical cells for the processing of colour vision. This feature appeared in studies of monkey striate cortex where most cortical cells showed no evidence of cone opponency, while many cells in the lateral geniculate nucleus did. Colour opponent cells also exist in cat retina and lateral geniculate nucleus[15,16], but they have never been detected in cat striate cortex, an area extensively studied. Again, the ratio of chromatic to achromatic selective cells seems to diminish in striate cortex. An important aspect of chromatic processing in monkeys is the existence of small, local areas called "blobs" [17], where cells with cone opponent behaviour are concentrated and therefore processing the same area of visual space. In murine cortex, most of the cells selective for UV stimuli were located at intermediate and caudal-lateral areas while cells selective for M-opsin stimuli were commonly located rostrally, which seems inappropriate for colour vision where comparisons of cone strengths must be determined within the same areas of visual space. It is interesting, however, that the three cone opponent cells we encountered were found in different areas, perhaps also reflecting small, local sites of chromatic processing.

We found two different types of neurons responding to our stimuli. One had little to no spontaneous activity and responded briefly and at high frequency to both wavelengths of stimulation and tended to have relatively large impulse amplitudes. The other class of neurons had more spontaneous activity, responded in a more prolonged way to our stimuli and usually had impulses of relatively small amplitude. Both of these cell types were recorded next to each other in most penetrations implying that they are similarly distributed. We found a similar dichotomy among retinal ganglion cells [5], but such a distinction has not been reported by other investigators of murine retinal ganglion cells [18]. Recordings from the murine lateral geniculate nucleus indicate the presence of burst-tonic responses from many cells, but there was no mention of a class of phasic cells [19]. There is some evidence of two classes of cells without orientation selectivity in murine striate cortex, one with conventional center/surround receptive fields and a second class with large fields best excited by fast moving stimuli [8,9]. More research will be needed to determine if two different cell systems target murine striate cortex.

## Conclusion

There were two varieties of cell responses. One type of cell, phasic cells, had little to no spontaneous activity and responded with a few impulses of large amplitude and high frequency either at the on and/or the off phase of the stimulus (42%). Another type of cell, which was more frequent (51%) had more spontaneous activity, impulses of smaller amplitude and tended to respond tonically.

Most cells in murine striate cortex receive excitatory inputs from both UV- and M-cones. A small fraction shows either strong selectivity for one or the other cone mechanism and occasionally cone opponent responses. The latter supports the hypothesis that mice may have some form of colour vision.

## Methods

### Animals

Recordings were made from the visual cortex of 48 C57BL/J6 mice anesthetized by intra-peritoneal injections of 60 mg/kg pentobarbital sodium (Nembutal, Abbot Laboratories, North Chicago, IL) or a combination of 100 mg/kg ketamine (Ketaset, 100 mg/ml, Fort Dodge Animal Health, Fort Dodge, IO) and 10 mg/kg xylazine (AnaSed, 20 mg/ml, Lloyd Laboratories, Shenandoah, IO). The experiments were carried out following the guidelines of the ARVO guidelines for animal experimentation.

### Recording and data acquisition system

The mouse's head was glued to a metal bar fixed to a stereotactic device. A 30-gage needle reference electrode was placed subcutaneously under the jaw; a similar ground electrode was placed on the back. The skin and bone overlying striate cortex was removed and the dura covered with hyaluronic acid (Healon, 10 mg/ml, AMO, Santa Ana, CA). The impulses of single neurons were detected by tungsten or 2 M KCl filled glass micropipette electrodes, the latter with resistances of 5–10 megohms. The tungsten microelectrode was introduced through the intact dura; glass micropipettes were introduced through a cut in the dura. The electrodes were slowly advanced through striate cortex by the micro-drive. Penetrations extended from the surface to a depth of about 1 mm as judged from the micrometer of an oil-coupled micro-drive. We usually made 4 or 5 penetrations in any one mouse monitoring responses with an oscilloscope, an audio amplifier and a digital data acquisition system. The responses were amplified and bandpass filtered (0.3 to 3 kHz) by a S-100 preamplifier (World Precision Instruments, Sarasota, FL) and stored on a Power Lab Chart system (AD Instruments, Colorado Springs, CO).

### Light stimuli

Both pupils were dilated using cyclopentolate eyedrops and the cornea was covered with hyaluronic acid (Healon, 10 mg/ml, AMO, Santa Ana, CA) to keep it moist. Either eye could be stimulated diffusely with either of two narrow band LEDs (370 nm, strong for UV cones and 505 nm, exclusively affecting M cones and rods). The energies of the stimuli produced by the two LEDs were measured with an IL-1700 radiometer (International Light Inc, Peabody, MA). The maximum energy of the 505 nm light emitting diode was 4.4 W/m^2^. The maximum energy produced by the 370 nm LED was 4.2 W/m^2^. These two LEDs appeared to produce relatively similar responses from most of the neurons encountered implying that there are similar numbers of these two cone types in murine retina as indicated by histological methods [14].

Eyes were light adapted with strong orange or white light to suppress rod activity. The contralateral eye was stimulated by either of these two wavelengths with light pulses of 100 to 500 milliseconds in duration, presented every 2.5 to 4 seconds. The ipsilateral eye could also be stimulated, but this was not routinely done.

### Recording strategy

201 penetrations were made into striate cortex of 48 mice following the maps of murine V1 [6,20,21]. In each penetration the impulse responses of a single neuron were compared to the two wavelengths. Sometimes there were multiple impulses from adjacent, responsive cells; in this case we concentrated on the unit of largest amplitude. We were able to monitor the waveform and amplitude of these impulses on an oscilloscope with a fast sweep speed in order to identify the particular unit being studied. We graded responses into five categories: cells excited exclusively by 505 nm; cells excited more strongly by 505 than 370 nm; cells affected equally; cells affected more by 370 than 505 nm and cells excited exclusively by 370 nm. We sometimes compared a cell's response to stimuli of reduced energies to determine the threshold of a cell's response.

We used a statistical software program (JMP 7.0, SAS Institute Inc., Cary, NC) to combine our estimates of UV-versus M-cone strength influencing each cell and the location of the cell in the striate cortex to produce a colour coded contour plot showing the topography of the cone inputs.

## Authors' contributions

Both authors contributed to all steps in this project.
